# A Pulse Coupled Neural Network Segmentation Algorithm for Reflectance Confocal Images of Epithelial Tissue

**DOI:** 10.1371/journal.pone.0122368

**Published:** 2015-03-27

**Authors:** Meagan A. Harris, Andrew N. Van, Bilal H. Malik, Joey M. Jabbour, Kristen C. Maitland

**Affiliations:** Department of Biomedical Engineering, Texas A&M University, College Station, TX, United States of America; Pennsylvania State Hershey College of Medicine, UNITED STATES

## Abstract

Automatic segmentation of nuclei in reflectance confocal microscopy images is critical for visualization and rapid quantification of nuclear-to-cytoplasmic ratio, a useful indicator of epithelial precancer. Reflectance confocal microscopy can provide three-dimensional imaging of epithelial tissue *in vivo* with sub-cellular resolution. Changes in nuclear density or nuclear-to-cytoplasmic ratio as a function of depth obtained from confocal images can be used to determine the presence or stage of epithelial cancers. However, low nuclear to background contrast, low resolution at greater imaging depths, and significant variation in reflectance signal of nuclei complicate segmentation required for quantification of nuclear-to-cytoplasmic ratio. Here, we present an automated segmentation method to segment nuclei in reflectance confocal images using a pulse coupled neural network algorithm, specifically a spiking cortical model, and an artificial neural network classifier. The segmentation algorithm was applied to an image model of nuclei with varying nuclear to background contrast. Greater than 90% of simulated nuclei were detected for contrast of 2.0 or greater. Confocal images of porcine and human oral mucosa were used to evaluate application to epithelial tissue. Segmentation accuracy was assessed using manual segmentation of nuclei as the gold standard.

## Introduction

Carcinomas, cancers of epithelial tissues that cover the external and internal surfaces of the body, account for more than 80% of all cancers [1]. Visual or endoscopic examination followed by invasive tissue biopsy and histopathology is the current standard of care for detection and diagnosis of carcinoma. The microscopic examination of fixed, sectioned, and stained tissue includes evaluation of morphologic and architectural alterations, including increased nuclear-cytoplasmic ratio (NCR), enlarged nuclei, cellular pleomorphism, and irregular epithelial stratification [2]. Many carcinomas are preceded by a premalignant stage, in which the development of cancer can be prevented if detected and treated successfully. However, the clinical presentation of these precancerous lesions, such as oral leukoplakia, Barrett’s esophagus, colon polyps, and actinic keratosis of the skin, can be widespread, multifocal, and/or diffuse. Furthermore, regions of premalignancy can be clinically indistinguishable from benign lesions, complicating selection of a representative site to biopsy for accurate diagnosis and staging.

In contrast to the physical sectioning of tissue required for histology, confocal microscopy achieves “optical sectioning” by spatially filtering light with a small aperture at the conjugate image plane of the microscope focus [3]. By detecting light from the focal plane and significantly rejecting out of focus light, confocal microscopy enables high resolution imaging in three dimensions of thick tissue. Recent advances in acquisition speed and miniaturization of confocal microscopes and endomicroscopes have enabled minimally-invasive real-time imaging of cellular and tissue features *in vivo* [4–10]. These features provide information comparable to histologic characteristics of the mucosa and submucosa without the tissue excision and processing required for histology. Confocal endomicroscopes have been implemented through working channels of conventional endoscopes and as independent instruments [4]. Beyond the current clinical use of confocal microscopy to detect neoplasia in gastroenterology and dermatology, confocal imaging is currently under investigation to improve early detection of cancer and precancer in a number of other organs [11–21].

Both fluorescence and reflectance confocal microscopy and endomicroscopy have been developed for tissue imaging [4]. While fluorescence confocal microscopy can provide high contrast images of cellular features, it requires administration of exogenous fluorescent dyes either topically or systemically to provide contrast. Reflectance confocal microscopy (RCM) exploits endogenous reflectance contrast produced by natural variations in the refractive index of cellular and tissue components [22,23]. Low concentration acetic acid (vinegar) may be applied to enhance contrast of cell nuclei and is already approved and routinely used in humans [24,25]. Use of near-infrared illumination in RCM allows deeper tissue penetration over one-photon fluorescence confocal, extending through the full thickness of the epithelium [23].

Segmentation of cell nuclei and quantification of NCR and nuclear size in RCM images of epithelial tissue can enable objective evaluation of tissue features for precancer detection [26,27]. If this data were provided rapidly or in real-time, the technique could be used to guide biopsy site selection and improve diagnostic yield. However, nuclear to background contrast can be low in RCM images, particularly in comparison to fluorescence confocal imaging with dye-based contrast agents. Furthermore, reflectance signal from the epithelium is not specific to cell nuclei. Cell borders, intracellular organelles such as the endoplasmic reticulum and mitochondria, melanin, and keratin all contribute to detected backscattered signal [23].

There is a need for automated cell nuclei segmentation to provide rapid image analysis of reflectance confocal microscopy and endomicroscopy images of epithelial tissue; however, the low contrast, non-uniform images confound the development of accurate segmentation algorithms [28,29]. Quantitative data may be obtained by tedious manual segmentation of nuclei. Although this method introduces inter- and intra-observer variability, it remains the gold standard for evaluation of segmentation accuracy.

Thresholding, a simple and commonly used segmentation algorithm, has proven to be useful in medical image processing [30]. This method relies on the pixel intensity of a region of interest and isolates the region based on whether a pixel is above a certain threshold value. Variations in region intensity are compensated for by calculating the optimal threshold for each individual image, known as Otsu’s method [31]. Otsu’s method is an algorithm that determines the threshold that minimizes the intra-class variance in an image, assuming that the image contains only two classes of pixels. However, the technique suffers when trying to segment regions of non-uniform intensity, a factor prevalent in confocal reflectance images. For instance, a free open-source software package, CellProfiler.org, is available for object segmentation using Otsu’s method; however, manual control of threshold values complicates rapid evaluation of images with varying contrast across a single image or images at multiple depths.

Other common segmentation techniques include the edge detection and watershed algorithms. Edge detection is a technique that attempts to identify features in the image through differentiation [32]. These features are identified through different filtering methods such as those by Canny, Sobel, or Prewitt [33–35]. However, attempts to identify and separate features are often confounded by image noise and regions of non-uniform intensity. The watershed segmentation algorithm has been successfully used in some confocal images [36], and is based on modeling the image as a topographical relief. The algorithm requires the use of markers to prevent over-segmentation of the image, which can be difficult to obtain automatically and may require manual methods.

Most segmentation algorithms reported in the literature were designed to segment higher contrast confocal images with fluorescently stained tissue [36–40]. Previous work done by Luck et al. introduced a segmentation algorithm by use of Gaussian Markov random fields (GMRF) for reflectance confocal endomicroscopy images of epithelial tissue [41]. The GMRF technique utilizes local pixels to estimate the actual grayscale value of a pixel. This generates regions of uniform intensity that can be segmented by morphological features such as size and eccentricity. The algorithm has been shown to be successful, detecting 90% of nuclei in a frame at a 14% error rate [41]. However, the algorithm suffers from over-segmentation in some images, resulting in a number of false-positives. Additionally, it is difficult to determine the number of fields required to make a good segmentation of each image.

Pulse coupled neural networks (PCNN) are derived from research on the mammalian visual cortex done by Eckhorn [42]. The network provides a useful biologically inspired tool for image processing. Each neuron represents a pixel on the image and is affected by the initial state of the pixel in the image, and the states of the surrounding neurons. The output of the network generates a series of temporal pulses, which can be used in many different image processing applications such as image segmentation or image fusion [43]. While the original PCNN model is strictly based on the neuron model created by Eckhorn, there are other networks specifically designed for image processing methods such as the intersecting cortical model or spiking cortical model (SCM) [44].

The algorithm introduced in this paper utilizes a PCNN, specifically the SCM, to fully automate the segmentation process. The algorithm is able to efficiently segment epithelial nuclei over varying depth below the tissue surface and output valuable quantitative information such as the nuclear-to-cytoplasmic ratio, number of objects segmented, average nuclear area, and standard deviations where appropriate.

## Materials and Methods

### Sample Preparation and Image Acquisition

#### Imaging of porcine oral mucosa

Normal porcine cheek tissue was acquired through the tissue sharing program at Texas A&M University (TAMU) which is designed to reduce the number of animals needed to accomplish research goals. Because the tissue was transferred from another study approved by the TAMU Institutional Animal Care and Use Committee (IACUC) after the animal was terminated, review of this work by the TAMU IACUC is not required. Following excision from the oral cavity, the buccal tissue was transported to the lab for imaging. Prior to confocal reflectance imaging, the sample was submerged in acetic acid for 1 minute to enhance nuclear contrast. The bulk sample was rinsed in a phosphate buffered solution and then placed on the inverted confocal imaging system with the buccal mucosal surface facing down towards the microscope objective.

Confocal reflectance images of the porcine buccal mucosa were acquired with 830 nm illumination using the VivaScope 2500 (Caliber I.D., Rochester, NY). This instrument is an inverted RCM designed to image unsectioned excised surgical specimens. Individual frame size provides a field of view (FOV) of 750 × 750 μm^2^ at a rate of 9 frames per second. Optical resolutions are 1.5 μm and 5 μm for lateral and axial planes, respectively. Images were acquired down to approximately 160 μm below the tissue surface using an infinity corrected, 0.85 numerical aperture (NA), water immersion objective lens. At this depth, reflectance signal is still detectable and the bases of rete ridges are prevalent; however, nuclei are no longer resolvable due to tissue scattering. A 3 × 3 array of images was captured at each depth to increase the total area imaged. To evaluate segmentation capability in images of different contrast and tissue features, images at four depths, approximately 8, 24, 40, and 64 μm below the surface, were analyzed. Images were cropped to a circular region with a diameter of 450 μm in order to reduce the range of focal plane depth due to field curvature. Nuclear objects were manually segmented by a single observer (K.M.) via visual recognition by applying nuclear masks to nuclei in a given frame. This manual segmentation was used as the gold standard in evaluation of the automatic segmentation algorithm. The objects’ size, distribution, and contrast were also used to create an image model.

#### Imaging of human oral mucosa

In order to demonstrate that the applicability of our approach is relatively independent of imaging system characteristics and epithelial tissue type, we applied the segmentation algorithm to images of excised human oral tissue acquired using a different RCM system. The detailed configuration of this system is described elsewhere [45]. Briefly, the illumination light, emitting at 810 nm, was raster scanned and focused through a water immersion microscope objective (40×, 0.8 NA). Light backscattered from the tissue was focused onto a pinhole before being detected by a photomultiplier tube detector. The field of view was measured to be ~625 μm diameter at the sample, with lateral and axial resolutions of 0.65 μm and 5 μm, respectively. Oral tissue collection and imaging protocols were approved by the Institutional Review Boards at Texas A&M University and TAMU—Baylor College of Dentistry, and written consent was obtained from study participants. The images presented here were obtained from a human tissue biopsy that was clinically diagnosed as inflammation and histopathologically diagnosed as gingival hyperplasia. RCM imaging was performed within 30 minutes following biopsy. Gauze soaked in acetic acid was applied for 1 minute prior to imaging. In comparison to porcine mucosa, we were able to image and observe discernable features relatively deeper (>300 μm) within the human oral tissue.

### Image Model of Epithelial Tissue

An image model was created in MATLAB to closely represent confocal images of epithelial tissue. Parameters such as nuclear size, density, and contrast were obtained directly from manually segmented confocal reflectance images of porcine buccal mucosa at various locations and depths. 750 circular objects with an area of 90 px^2^ (corresponding to ~8 μm diameter nuclei) are randomly distributed without overlap in a set of six 1000 × 1000 px^2^ images. The ratio of average nuclear intensity to average background intensity (nuclear to background contrast ratio) was varied from 2.6 to 1.6 by decreasing the signal from the nuclear objects and increasing the signal from the background to simulate the loss of contrast with increasing imaging depth in tissue. Each object is modeled with a Gaussian spatial distribution of pixel intensity to adequately represent the nuclear appearance. Each object’s peak signal was determined using a histogram of intensities of manually segmented nuclei from depths with comparable nuclear to background contrast ratio. The background signal was modeled as Gaussian white noise with mean and variance based on intensity variation in cytoplasmic signal bordering nuclei in manually segmented images at depths with comparable nuclear to background contrast ratio. The background of the image model did not include tissue features such as cell borders and areas of high keratinization.

### Spiking Cortical Model Algorithm

**Fig 1:**
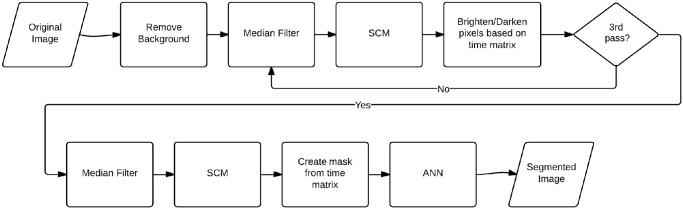
Flowchart showing main steps of the automated SCM segmentation algorithm for segmenting nuclei in RCM images of epithelial tissue.

**Fig 2:**
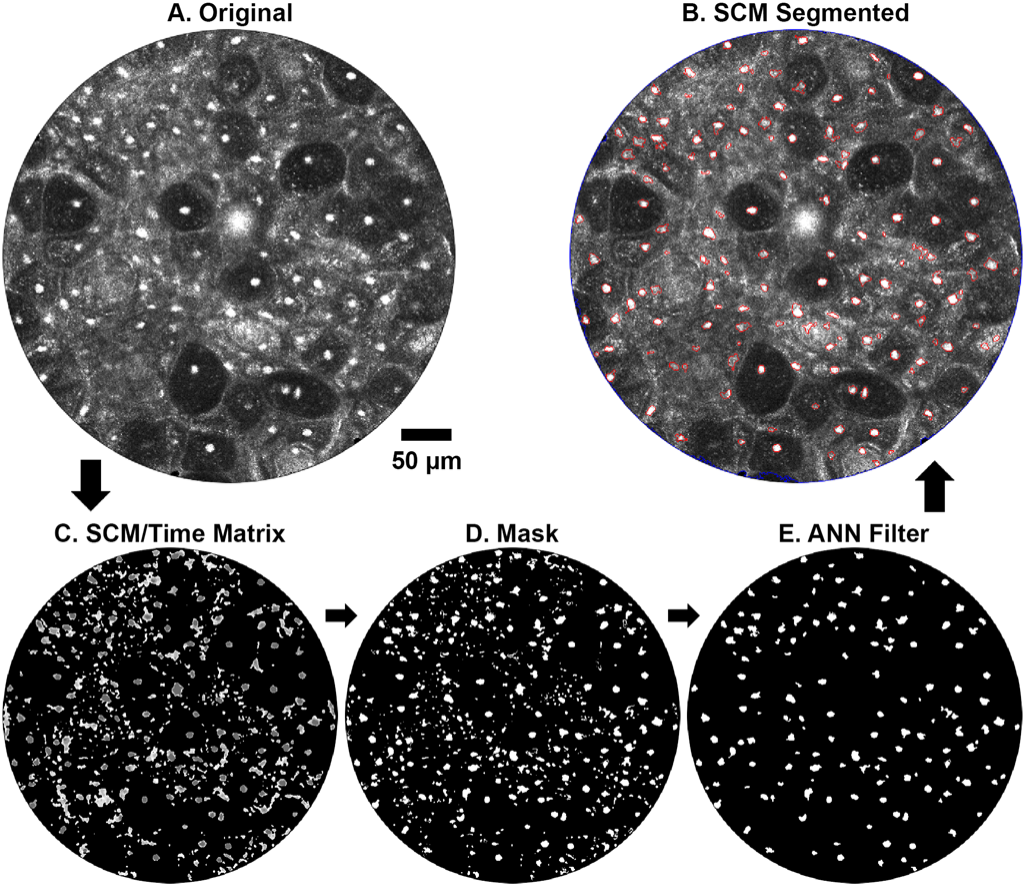
Image steps of SCM segmentation algorithm. (A) Original confocal image of porcine buccal mucosa showing range of nuclear to background contrast. (B) SCM segmentation of (A). (C) Output of final SCM filter showing time matrix of pulse outputs. (D) Segmentation mask obtained from the time matrix. (E) Output of the ANN classifier defining the segmented objects in (B).

The automated PCNN based algorithm was written in MATLAB (The MathWorks, Inc., Natick, Massachusetts, United States) and is available at the online Zenodo database (https://zenodo.org/record/12804) [46]. The nuclear-to-cytoplasmic ratio, area of segmented nuclei, and the total number of objects segmented were recorded and compared to manual segmentation. Object- and pixel-based sensitivity and pixel-based specificity were calculated using the manually segmented images as the gold standard. For object-based sensitivity, an object was considered a true positive if any of the pixels within an object were correctly classified. All other missed objects were considered false negatives. For pixel-based specificity, the number of true negative pixels was counted as the active FOV less the total number of true positive, false positive, and false negative pixels. The flow chart for the SCM algorithm can be seen in Fig 1. The main steps shown in the chart are described in detail below. Fig 2 illustrates various steps in the algorithm during the segmentation process of an example confocal image (A). The SCM algorithm takes approximately 20 seconds to process a single confocal image, performed on a laptop computer with 2.3 GHz processing speed and 16 GB of RAM.

#### Background removal

Occasionally, the tissue may not fill the entire imaging FOV. If the active FOV is not well-defined, NCR calculations using the entire FOV may be erroneously low. A threshold algorithm is utilized to remove the background of the image leaving the active FOV. This was accomplished by assuming the background is composed of a large contiguous area of dark pixels distinct from the foreground (i.e. the tissue). A scaled threshold for the background was calculated using Otsu’s method [31], which provides an optimal division between the foreground and background of each image. Subsequently, an area filter was applied to remove the background from the image. After removal of the background pixels, the area of the foreground was calculated for use in the NCR calculation.

#### Pulse coupled neural network

Image filtering and segmentation is carried out by the SCM developed by Zhan, et al [47], which models pixels in an image as neurons in an interconnected neural network. The SCM is a biomimetic algorithm and a simplified variant of the original PCNN visual cortex model. The model itself is composed of three equations: an internal activity function, a neural output function, and a dynamic threshold function. The matrix created by the output function is the only result that is examined. The threshold and internal activity functions are hidden and only used to calculate the output. Finally, a time matrix is a single composite image created by these outputs that records the pulse time of each neuron run through the SCM. The functions compose an abstract representation of a biological visual model that separates various “features” of an image into different outputs separated temporally. Here, we define a feature as a set of pixels of similar intensity grouped spatially. Image filtering is done by reducing the amount of features present within the image, while the features of interest (i.e. nuclei) are isolated in segmentation. The extracted features depend on a number of parameters, *f*, *g*, *W*, and *h*, as described below and in Chen, et al. [48].

A neuron in the SCM model is defined as these three equations applied to a single pixel within the image. The variables *i* and *j* denote the position of the neuron of interest in the image, while *k* and *l* define the positions of the neighboring neurons relative to the current neuron. The internal activity function takes an input image and forwards it into the neuron layer:

$$U_{ij}\left(n\right)=fU_{ij}\left(n-1\right)+S_{ij}\sum_{kl}W_{ijkl}Y_{kl}\left(n-1\right)+S_{ij}$$ (1)

where *S*_*ij*_ is the input image, *U*_*ij*_ is the internal activity of the neuron, *Y*_*kl*_ is the neuronal output of the neighboring neuron, and *n* defines the current iteration of the network. The parameter *f* is the decay coefficient for the internal activity function, which affects the temporal spacing of features. The parameter *W*_*ijkl*_ is the weight matrix determines the connections between neurons, or the association strength of neighboring pixels within a feature [48].

The neural output function compares the internal activity of the neuron to its current threshold:

$$Y_{ij}(n)=\{\begin{array}{c} 1,U_{ij}(n)>E_{ij}(n)\\ 0,otherwise \end{array}$$ (2)

where *Y*_*ij*_ is the neuronal output and *E*_*ij*_ is the dynamic threshold of the neuron as defined in Equation 3. The threshold function is calculated by addition of the previous threshold with the neuronal output:

$$E_{ij}(n)=gE_{ij}(n-1)+hY_{ij}(n)$$ (3)

The parameters *g* and *h* are defined as the decay coefficient and amplitude for the threshold function, respectively. Both parameters determine the precision of intensities for each feature [48].

Through trial and error, *f*, *g*, and *h* were set to 0.928, 1.078, and 1.4, respectively. The parameter *W*_*ijkl*_ was set to [0.0125, 0.025, 0.0125; 0.025, 0, 0.025; 0.0125, 0.025, 0.0125]. Once these tunable parameters were optimized using training data from porcine buccal mucosa, they were kept constant for segmentation of all porcine and human tissue images. While automated methods exists for setting PCNN parameters [48], manually setting the parameter values for the network provided more desirable results, such as maximizing true positives while reducing false positives. The final values were chosen so that the algorithm was tuned to provide an optimized output for both sensitivity and specificity. The parameters could be modified to prioritize sensitivity over specificity, for example, if a specific application warranted it. However, all tunable parameters depend on each other and affect output results, complicating this parameter tuning.

#### Time matrix

The network was modified such that each neuron representing a pixel could only output once. Each successive neuronal output was labeled by iteration number, generating a time matrix as seen in Fig 2(C). The time matrix is a composite image that combines the pulse outputs of the SCM, and organizes the pixels in the image based on similar intensity [47]. Since brighter elements of the image (e.g. bright pixels, nuclei, etc.) are stored in earlier iterations and darker elements (e.g. background, dim artifacts, out of focus objects etc.) are stored later, the time matrix can label the image according to the pulsing order. Here, we use the time matrix to 1) filter and 2) obtain the segmentation mask, Fig 2(D).

Since darker elements of the image are stored in later iterations of the network, only the first 6 output iterations of the network are analyzed. The size and eccentricity values were measured for each object within each output iteration. Using this information, the pixel intensity values in the original image were brightened or darkened with gamma corrections. For the earlier iterations, large areas were lowered in intensity to darken the intensity of large saturated areas. For later iterations, small, round areas were raised in intensity to brighten darker nucleus shaped objects.

Following successive PCNN filtering, a final time matrix was generated to produce a segmentation mask. An initial mask was first created by taking the regional minima of the time matrix. This process captures the central area of each nucleus, but leaves out the periphery. By “growing” the initial mask by adding successive iterations, a segmentation mask was obtained. Each iteration was added to the segmentation mask until the object passed a set area and eccentricity limit.

#### Artificial neural network classifier

Following the segmentation done by the PCNN, an artificial neural network (ANN) classifier was made using the built-in MATLAB Neural Network Toolbox™. By inputting a set of examples to this classifier, we train the classifier to remove objects that are more likely to be false positive nuclei based on a set of features. Using a database of objects created by manual segmentation, the network was trained by an 8-dimensional feature vector which included area, eccentricity, extent, solidity, foreground intensity and standard deviation, and background intensity and standard deviation. Out of the 36 total images, two images were randomly selected at each depth with no overlapping lateral position for all depths. The database of approximately 1800 objects in these 8 images was randomly divided into three sets: 70% as a training set, 15% to validate that the network is generalizing and to stop training before overfitting, and 15% as an independent set to test for network generalization. After creating the neural network classifier with these 8 images, it was applied to the remaining 28 images. All reported results and images in this paper are from the 28-image dataset and do not include the 8-image training data. For the image in Fig 2(A), a finalized segmentation output of nuclear objects, Fig 2(B), was obtained after removal of the ANN classified objects from Fig 2(E).

#### MATLAB output and user interface

The MATLAB regionprops function to measure properties of image regions was applied to the image mask in order to generate information about nuclear area, NCR, and the number of objects detected in the image. The mean and standard deviation for nuclear area was calculated for the image. The NCR was calculated by taking the total area of the objects present in the image and dividing by the remaining area in the FOV. Fig 2(B) shows an image output generated by the SCM algorithm which includes a blue border designating the segmented active FOV and red borders around each segmented object.

The MATLAB GUIDE tool was used to build a user interface for the algorithm. The interface enables the quick processing of multiple images, as well as preview and batch save capabilities. In addition, the interface enables the export of the information generated for each image as a Microsoft Excel spreadsheet file and lists the data accordingly for each analyzed image.

## Results and Discussion

### Image Model

**Fig 3:**
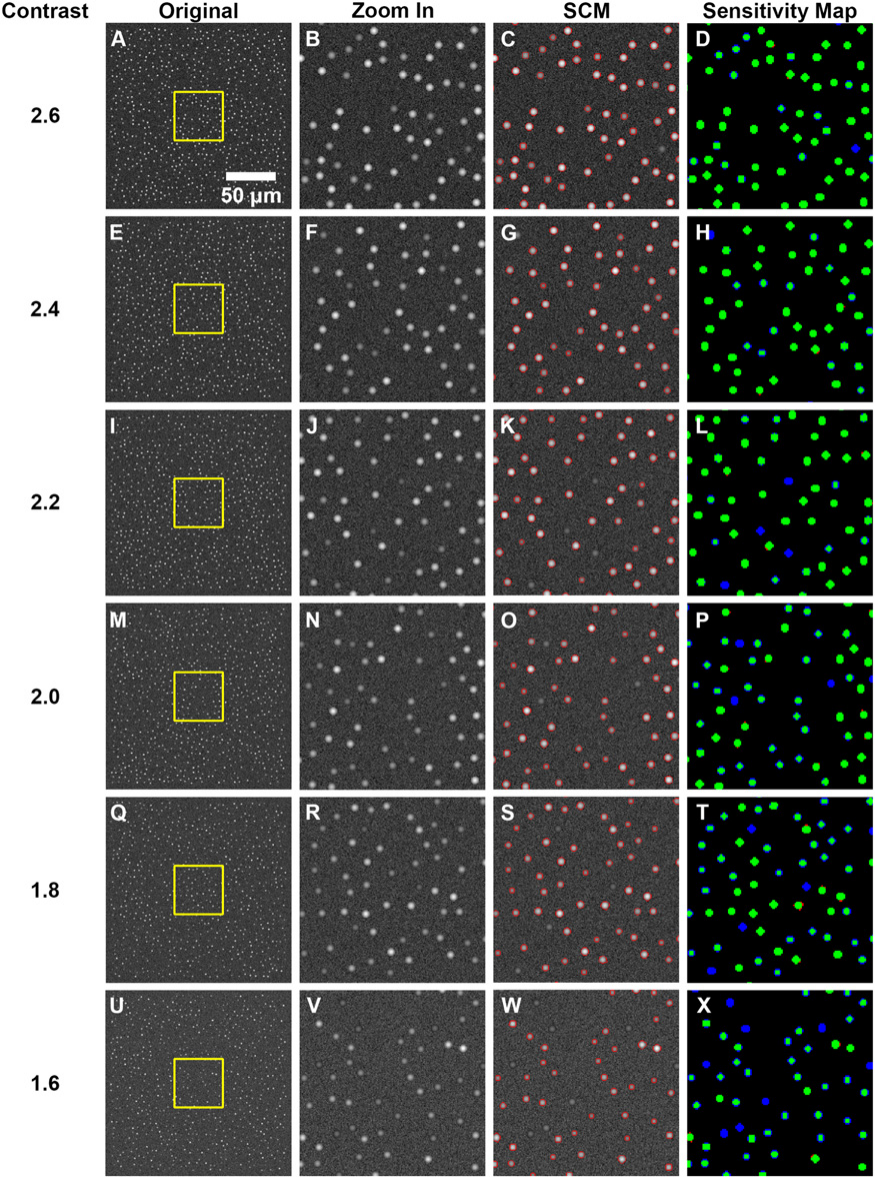
SCM segmentation of confocal image model with sensitivity evaluation. 750 × 750 μm2 field of view confocal image model with nuclear to background contrast (A) 2.6, (E) 2.4, (I) 2.2, (M) 2.0, (Q) 1.8, and (U) 1.6. (B), (F), (J), (N), (R), (V) 50 × 50 μm2 field of view zoom in of yellow box from (A), (E), (I), (M), (Q), and (U), respectively, showing nuclear object detail. (C), (G), (K), (O), (S), and (W) SCM segmentation and sensitivity map comparison to objects in the original model images of (B), (F), (J), (N), (R), and (V), respectively. Green pixels (true positives), blue pixels (false negatives), and red pixels (false positives).

The SCM automated segmentation algorithm, including the ANN classifier trained on objects in confocal images of epithelium, was first applied to the image model of epithelial tissue to evaluate the limitations of the algorithm. The segmentation performance was assessed based on sensitivity (true positive rate) and specificity (true negative rate) for each contrast value or nuclear to background ratio. Sensitivity was calculated using both object and pixel based methods. Specificity was calculated by pixel based method only due to the inability to quantify true negative objects. SCM analysis of the image model is shown in Fig 3. Contrast decreases from 2.6 to 1.6 moving down each column. The original simulated images shown in the first column are 1000 × 1000 px^2^ FOV containing 750 objects distributed randomly without overlap. The yellow box indicates the location of the zoomed in 250 × 250 px^2^ FOV regions featured in the second column. The third column shows the SCM segmentation of the zoom in area. The fourth column is a sensitivity map depicting the accuracy of the SCM algorithm to detect simulated nuclear objects with varying contrast. Green pixels on the sensitivity map indicate true positives, blue pixels are false negatives, and red pixels are false positives.

### Porcine Buccal Mucosa Images

**Fig 4:**
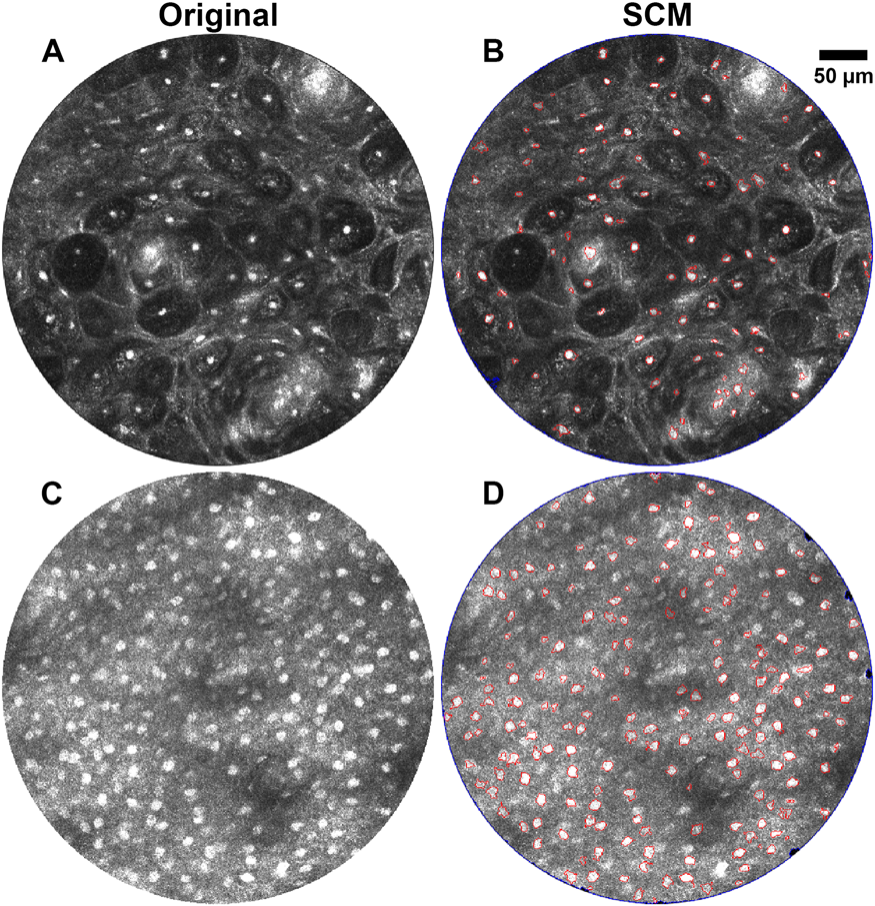
Comparison of SCM segmentation algorithms on confocal images of oral mucosa with high and low contrast. Original confocal images of porcine buccal mucosa with (A) high and (C) low nuclear to background contrast. (B) and (D) SCM segmentation of (A) and (C), respectively.

Automated segmentation analysis was performed on 28 confocal images of excised porcine buccal mucosa at four depths spanning the surface to 60 μm in depth. Example SCM segmentation results for two images are shown in Fig 4. The image in Fig 4(A), obtained approximately 20 μm below the tissue surface, represents a high contrast, easily segmented image from the superficial layer in the epithelium. The image has 79 manually segmented objects and an average object to background contrast of 2.26. The image in Fig 4(C), obtained approximately 60 μm below the surface, demonstrates relative difficulty in segmenting low contrast images. This image has 259 manually segmented objects and an average contrast of 1.51. SCM segmentation is shown in Fig 4(B) and 4(D). The high contrast image in Fig 4(A) had the highest SCM segmentation sensitivity, with an object based sensitivity of 94%, pixel based sensitivity of 84%, and pixel based specificity of 99%. For the low contrast image in Fig 4(C), SCM segmentation had object based sensitivity of 70%, pixel based sensitivity of 61%, and pixel based specificity of 98%.

**Fig 5:**
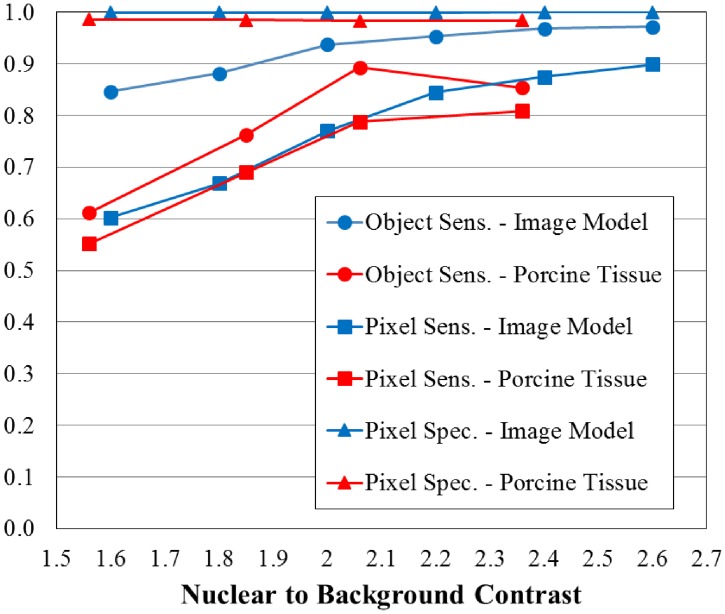
Effect of nuclear to background contrast on accuracy of SCM segmentation. Object-based sensitivity (Object Sens.), pixel-based sensitivity (Pixel Sens.), and pixel-based specificity (Pixel Spec.) are plotted for SCM segmentation of the simulated image model and captured images of porcine buccal mucosa as a function of contrast. Increasing depth below the surface of mucosal tissue corresponds to decreased contrast.

The plot in Fig 5 illustrates the SCM segmentation results of the confocal image model and the porcine buccal mucosa tissues with respect to image contrast. With increasing image contrast, both object-based and pixel-based sensitivities improve. Because the image model simulates only nuclear objects and not other cellular features that scatter light, there were very few false positive pixels and numerous true negatives. Therefore, the specificity for all confocal image model figures is practically 100%. The highest object-based sensitivity measured for the confocal image model was 97% for a nuclear to background contrast of 2.6. Whereas, the highest object-based sensitivity measured for the porcine tissues was 89% with an image contrast of 2.06. Pixel-based sensitivity is less than object-based sensitivity primarily due to undersegmentation of objects yielding more misclassified pixels within detected objects. The specificity does not vary significantly due to the disproportionately large number of pixels in the active FOV in comparison to the false positive pixels.

Table 1 summarizes the performance of the SCM segmentation algorithm over 28 images of porcine tissue. A total of seven adjacent images were analyzed at each of four depths. The table includes image properties such as average object to background contrast and total number of manually segmented objects at each depth below the tissue surface. The number of true positive (TP) and false positive (FP) objects, and percent error are presented for SCM segmentation. A pixel-based F-measure value, where$F1=\frac{2\times sensitivity\;\times specificity}{\left(sensitivity+specificity\right)}$, is also reported as a means to evaluate accuracy of the segmentation algorithm based on both sensitivity and specificity [49].

**Table 1:** SCM segmentation of confocal images of porcine tissue.

Image Properties			SCM				
Depth [μm]	Contrast	# Objects	True Positives	False Positives	% Error Sensitivity (object)	% Error Sensitivity (pixel)	F-Measure
**7.93**	2.36	554	473	271	14.62%	19.14%	0.89
**23.78**	2.06	749	669	193	10.68%	21.22%	0.87
**39.63**	1.85	1167	889	63	23.82%	31.01%	0.81
**63.40**	1.56	1479	902	42	39.01%	44.86%	0.71

**Fig 6:**
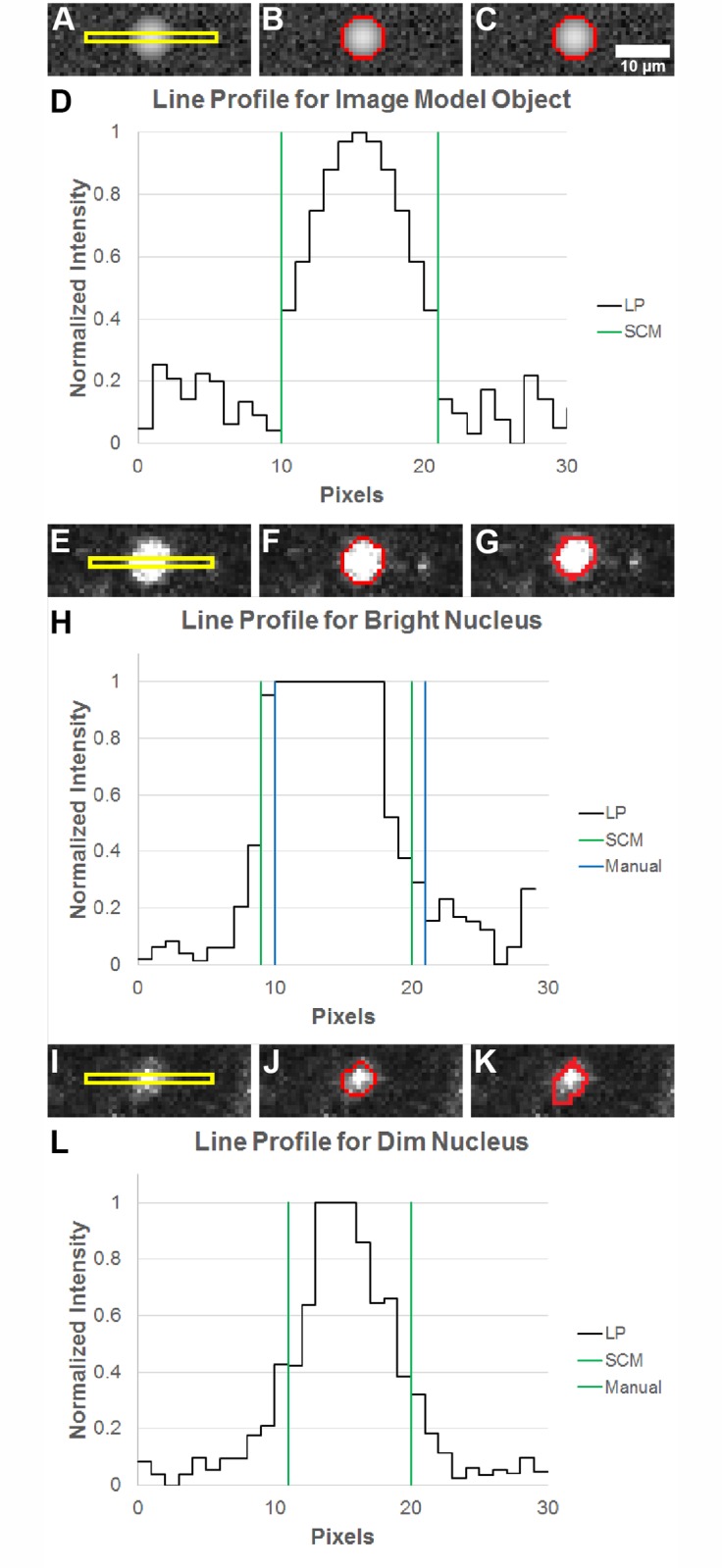
Line profile plots to compare SCM and manual segmentation. (A) Representative image model object, (B) SCM segmentation of selected object, and (C) actual object border. (D) Normalized line profile (LP) plot for line indicated by the yellow box in (A). (E) Bright and (I) dim nuclei, (F) and (J) SCM segmentation, and (G) and (K) manual segmentation. (H) and (L) Normalized LPs with segmentation borders identified for SCM and manual segmentation. Note in (L) that SCM and manual segmentation overlap.

To further compare segmentation by SCM to manual segmentation, normalized line profiles through two nuclei from the image in Fig 4(A) are shown in Fig 6 as well as a line profile through an image model object. The original image of an object from an image model figure is shown in Fig 6(A). A line profile though the object is shown in Fig 6(D) where the green lines represent the SCM segmentation, Fig 6(C), which overlaps on the designated object border as seen in Fig 6(B). The original images of a bright, well-resolved nucleus and a dim nucleus that is not easily resolved are shown in Fig 6(E) and 6(I), respectively. Segmentation of the well-resolved nucleus, Fig 6(H), illustrates that SCM segmentation tends to have a tight fit around nuclei, similar to manual segmentation. The intensity plot for a nucleus that is not as well-resolved is shown in Fig 6(L). Here, the green lines represent both SCM and manual segmentation, which overlap for this orientation of the line profile of the nucleus. As seen in these plots, manual segmentation and SCM segment nuclei around the half maximum point.

Table 2 summarizes SCM segmentation of confocal images of epithelial tissue in comparison to manual segmentation. The total number of objects segmented is listed per depth. The greater number of objects segmented near the surface by SCM may be attributed to false positives from non-nuclear image features. Average NCR, nuclear area, and nuclear diameters are also shown by depth below tissue surface. Because of the irregular shape of nuclear objects, average diameter is calculated from the average area. As expected for epithelial tissue, the number of segmented objects increases with image depth; however, the number of objects segmented with SCM does not increase at the same rate as manual segmentation. This reduced sensitivity, due to low nuclear to background contrast, results in underestimation of NCR with depth.

**Table 2:** Comparison of SCM segmented objects to manually segmented objects.

Image Properties		SCM				Manual			
Depth [μm]	Contrast	# Objects	NCR	Nuclear Area [μm^2^]	Diameter [μm]	# Objects	NCR	Nuclear Area [μm^2^]	Diameter [μm]
**7.93**	2.36	744	0.0345	49.11	7.91	554	0.0223	43.53	7.44
**23.78**	2.06	862	0.0452	54.44	8.33	749	0.0354	49.07	7.90
**39.63**	1.85	952	0.0556	60.06	8.74	1167	0.0597	52.11	8.15
**63.40**	1.56	944	0.0623	68.28	9.32	1479	0.0912	61.03	8.82

**Fig 7:**
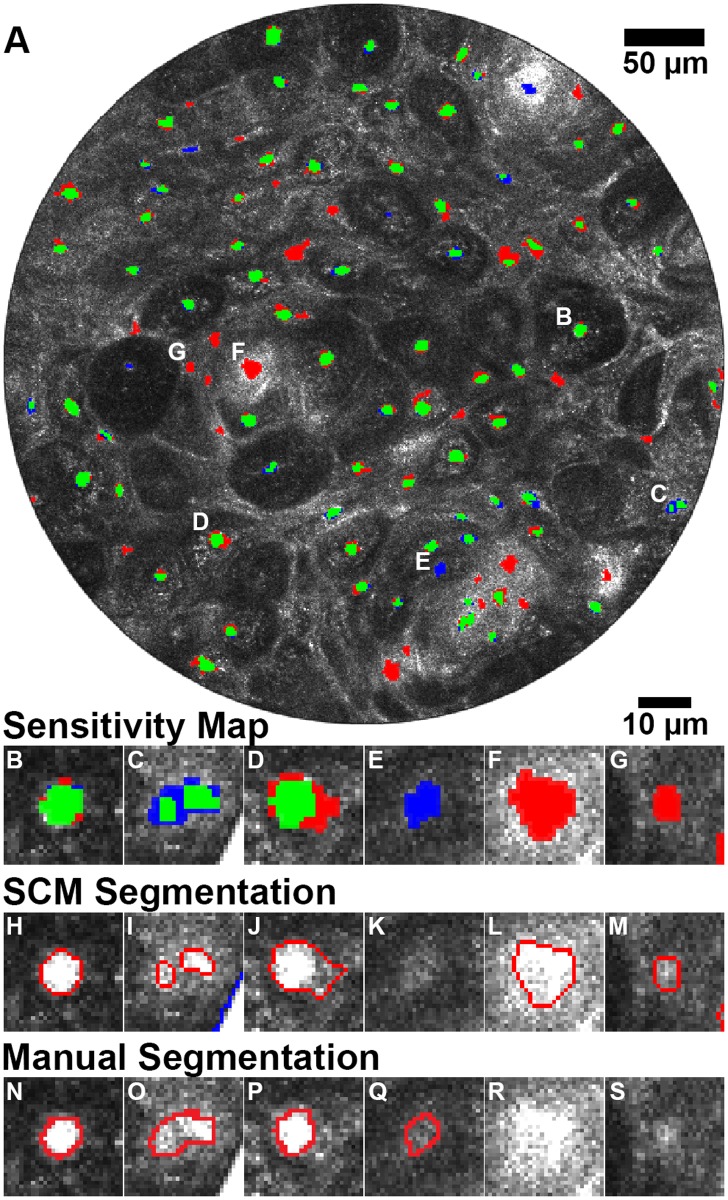
Evaluation of SCM segmentation performance. (A) Sensitivity map demonstrating true positives (green), false negatives (blue), and false positives (red). Objects of interest are labeled (B-G). Zoom-in of example nuclei demonstrating (B) excellent segmentation, (C) and (D) oversegmentation, (E) false positive, (F) image artifact false positive, and (G) false positive potentially missed by manual segmentation. (H-M) SCM and (N-S) manual segmentation of (B-G).

To illustrate the performance of SCM segmentation of individual nuclei, Fig 7 depicts examples of various segmentation scenarios that affect quantitative output of the algorithm. The confocal image from Fig 4(A) is seen in Fig 7(A) with a sensitivity mask overlaid on top. Green areas indicate a true positive match to the manual segmentation. Blue indicates a false negative, a nuclear object that was not segmented by SCM but was manually segmented. Red areas are designated as false positive, or objects that SCM detected but were not segmented manually. Fig 7(A) is labeled with the locations of the nuclei detailed here and shown in Fig 7B–7G. Fig 7H–7M are the SCM segmentation corresponding to the sensitivity maps of Fig 7B–7G, respectively. Fig 7N–7S are the corresponding manual segmentation. The sensitivity map, Fig 7(B), shows an example of excellent segmentation by SCM as compared to manual segmentation. Fig 7(C) is an example of over-segmentation by SCM or excessive nuclei splitting due to low pixel intensities. The object was manually segmented as a single object because it is unlikely, but possible, that two nuclei would be positioned so close together within this superficial epithelial layer. Another example of over-segmentation also likely caused by low pixel intensities is shown in Fig 7(D), where the SCM algorithm segmented the object larger than the manual segmentation. It is possible that some nuclei may not be as well resolved due to their depth position relative to the focal plane, resulting in reduced intensity. Fig 7(E), a false negative, demonstrates this, showing the limitations of SCM segmentation. False positives can occur for many reasons such as tissue and image artifacts and, possibly, nuclei missed by manual segmentation. Fig 7(F) is an example of a false positive from a rete ridge or keratin pearl in the oral epithelial tissue. Fig 7(G) shows an object that was segmented by SCM, but not by manual segmentation. It appears to be the appropriate size of a nucleus, but was not manually segmented because of its location within the tissue.

### Human Oral Mucosa Images

**Fig 8:**
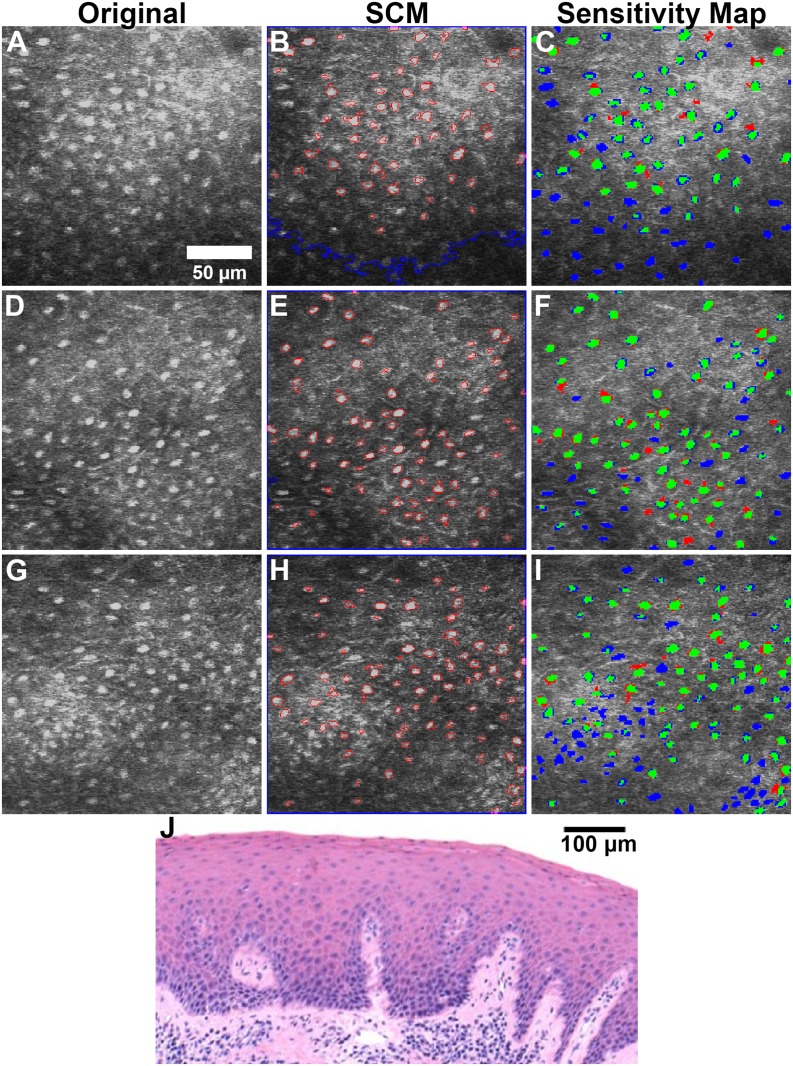
SCM segmentation of confocal images of oral mucosa at various depths. Original confocal images and SCM segmented images of inflamed human buccal mucosa at approximately (A, B) 90, (D, E) 180, and (G, H) 270 μm below tissue surface. True positives (TP), false negatives (FN), and false positives (FP) quantified for these depths were (C) 54 TP, 34 FN, 3 FP; (F) 72 TP, 26 FN, 7 FP; (I) 80 TP, 53 FN, 1 FP. Histology image (J) shows full epithelium. Images have been cropped from media file to restrict depth.

To demonstrate the ease of applicability of the SCM nuclear segmentation algorithm to images acquired from other tissue types and using different RCM systems, human oral mucosal tissue was imaged with a different RCM system [45]. A total of 9 confocal images spanning a range of depth from 60 μm to 300 μm within the human oral tissue were selected for validation of the SCM algorithm. These images were taken from a tissue biopsy suspected of inflammation and later classified by histopathology as gingival hyperplasia, a benign lesion. Fig 8 shows sections of the representative original and the corresponding segmented images from varying depths within the tissue. Note that in comparison to the porcine oral mucosa, overall slightly lower level of contrast was observed across the human oral epithelium. The object-based and pixel-based sensitivity values varied from ~50% to 73% and ~40% to 62%, respectively, and unlike images of porcine mucosa, without any correlation to the depth of imaging. The pixel-based specificity did not vary significantly and was over 98% in all instances.

It is worth noting that, in general, the values of sensitivity were lower in comparison to that of porcine tissue. This can be attributed to a number of reasons. The histology corresponding to the tissue biopsy was interpreted as hyperplasia which is typically marked by a benign proliferation of cells. This can be observed in the images throughout Fig 8 wherein the nuclear density is higher than that of Fig 4(A) and somewhat similar to that of Fig 4(C) (which is near the basement layer in porcine mucosa). Such nuclear crowding can result in a decrease in forward scattered light, as predicted by the theory of light scattering by densely packed structures [50]. A direct consequence of this is loss of contrast in the corresponding regions within the images. In addition, as can be seen in the histology section in Fig 8(G), the epithelial thickness varies from less than 100 μm (in the areas of rete ridges) to over 300 μm, and islands of inflammatory cells are observed to penetrate into the epithelium. Both the interface of rete ridges and of these islands manifest as significant heterogeneities within the refractive index of the medium and may result in an increase in the backscattered light, as can be noticed in the lower left quadrant of Fig 8(E). Such variation in both contrast and levels of backscattered light across a single image presents a limitation of our SCM algorithm, and results in a decrease in the number of segmented objects and, consequently, more false negatives.

Another possible factor towards explanation of such behavior is that in order to image visibly at the depths of >300 μm within tissue, the optical power at the sample was kept relatively high and constant, and hence less optimal for more superficial layers of the tissue. This effect can be seen in the accompanying media file (S1 Video) wherein areas within the images from approximately the top one-third of the epithelium are saturated, the middle one-third exhibit better segmentation, and the lower one-third thickness shows loss of contrast simply due to imaging at greater depths within tissue. Accordingly, the sensitivity values in the images from the middle one-third of the tissue are the highest and monotonically drop off towards either side.

It is worth noting that although manual segmentation results were considered as a benchmark for quantifying SCM algorithm performance, such a benchmark itself is prone to both intra-observer and inter-observer variation. For instance, differences in display output settings (hue, contrast, brightness, etc.) vary between display screens (monitors and television) of different makes and models, and can potentially affect the ability of a reader to segment the same image consistently. Inter-observer variation in manual image segmentation can have an even more profound impact since the training and understanding of the reader as to what area constitutes a cell nucleus is rather subjective. In order to quantify this effect, the pig tissue images were segmented by multiple readers (MH and AV in addition to KM). The average area of segmented nuclei was under- and over- estimated by MH and AV in comparison to KM by approximately 9% and 11%, respectively, highlighting the impact of inter-observer variation. Thus, such limitations should be kept under consideration when estimating the accuracy of any image segmentation algorithm. A potential alternative approach for establishing a gold standard would be to use a nuclear stain, such as DAPI, in fluorescence confocal microscopy to identify nuclei in *ex vivo* tissue samples. One-photon fluorescence has better resolution and reduced penetration depth in comparison to RCM; however, this would be an effective method for localization of nuclei in superficial epithelium. Although using exogenous contrast agents in humans is still limited for *in vivo* imaging, the use of fluorescent agents to enhance contrast in epithelial tissues has shown many benefits [4].

## Conclusion

Segmentation of nuclei in RCM images with low nuclear to background contrast is a challenge, particularly for fully automated algorithms. We have presented an automated PCNN nuclear segmentation algorithm based on the spiking cortical model. The segmentation accuracy was evaluated using an image model and confocal images of porcine oral epithelial tissue with varying nuclear to background contrast. The algorithm was further validated on RCM images obtained from human oral tissue using a custom-built imaging system. Although segmentation accuracy degrades with reduced contrast and increasing image depth in tissue, automated segmentation of nuclei is significantly faster than manual segmentation, enabling rapid evaluation of tissue properties such as NCR and nuclear size.

## Supporting Information

S1 Video Automated axial scan of inflamed human buccal mucosa. SCM segmentation of inflamed human buccal mucosa is shown spanning a depth of approximately 400 μm through the oral epithelium. Nuclear borders are identified with a red outline and field of view considered for segmentation is outlined in blue. Images cropped from this media file are shown in Fig 8.
(AVI)
